# Alterations in chromatin accessibility during osteoblast and adipocyte differentiation in human mesenchymal stem cells

**DOI:** 10.1186/s12920-022-01168-1

**Published:** 2022-01-31

**Authors:** Jianyun Liu, Lijun Gan, Baichen Ma, Shan He, Ping Wu, Huiming Li, Jianjun Xiong

**Affiliations:** Jiangxi Provincial Key Laboratory of Systems Biomedicine, Jiujiang University, Jiujiang, 332000 China

**Keywords:** MSC, ATAC-seq, RNA-seq, Adipogenic, Osteogenic

## Abstract

Although differential expression of genes is apparent during the adipogenic/osteogenic differentiation of marrow mesenchymal stem cells (MSCs), it is not known whether this is associated with changes in chromosomal structure. In this study, we used ATAC-sequencing technology to observe variations in chromatin assembly during the early stages of MSC differentiation. This showed significant changes in the number and distribution of chromosome accessibility at different time points of adipogenic/osteogenic differentiation. Sequencing of differential peaks indicated alterations in transcription factor motifs involved in MSC differentiation. Gene Ontology (GO) and pathway analysis indicated that changes in biological function resulted from the alterations in chromatin accessibility. We then integrated ATAC-seq and RNA-seq and found that only a small proportion of the overlapping genes were screened out from ATAC-seq and RNA-seq overlapping. Through GO and pathway analysis of these overlapped genes, we not only observed some known biological functions related to adipogenic/osteogenic differentiation but also noticed some unusual biological clustering during MSC differentiation. In summary, our work not only presents the landscape of chromatin accessibility of MSC during differentiation but also helps to further our understanding of the underlying mechanisms of gene expression in these processes.

**Supplementary Information:**

The online version contains supplementary material available at 10.1186/s12920-022-01168-1.

## Introduction

Osteoporosis, involving reductions in bone mass, microstructure reduction, and increased bone fragility and fracture, is a global public health problem [1]. According to the classical view, the occurrence of osteoporosis is related to increased osteoclast production and activity, resulting in the loss of bone mass and the decline of bone quality [2]. Recent studies have confirmed that an imbalance in the differentiation of bone marrow-derived mesenchymal stem cells (MSCs) into osteoblasts (OBs) and adipocytes (ADs) is a key factor in osteoporosis [3, 4].

MSCs are characterized by strong self-renewal abilities and can differentiate into different cell types [5]. The direction of differentiation is reflective of fundamental alterations in the gene expression pattern promoting the formation of adipocytes or osteoblasts. In in vitro culture, adipogenic/osteogenic inducers are commonly used to induce MSCs directional differentiation. After 14–21 days of treatment, a significant alteration in gene expression was observed to promote the differential differentiation and maturation of MSCs into adipocytes or osteoblasts [6, 7].

In eukaryotes, modulation of gene expression may occur at the levels of transcription, post-transcriptional regulation, translation, and post-translational modification. Due to the rapid development and application of next-generation sequencing, numerous investigations into the transcriptomes of adipocytes or osteoblasts derived from MSCs have been undertaken, providing a suitable basis for studying the mechanism of MSC differentiation [8–11]. However, the transcriptional activation mechanisms of many genes are still unclear.

Epigenetics dynamically regulates transcription and plays a crucial role in cellular functioning [12]. Assays for transposase accessible chromatin using sequencing (ATAC-seq) is an epigenetic technique used to identify the accessibility of chromatin [13]. Obtaining the location of the accessible region and the active regulatory sequence on chromatin helps to infer the possible binding of transcription factors and their actions in the whole genome [14]. Here, we aimed to investigate variations in chromatin accessibility in the early stage of MSC differentiation, integrating this with RNA-seq. This will provide a new entry point for exploring MSC differentiation and will help to identify more effective target genes for controlling osteogenic/adipogenic directional differentiation.

## Materials and methods

### hMSCs culture

Two strains of human primary cultured MSCs were acquired from the respective bone marrow of a 21-year-old and a 22-year-old healthy male. The isolation, characterization, culture, and storage of the MSCs were conducted as previously described [15]. For adipogenic differentiation assays, sixth-generation MSCs were incubated with a stimulation cocktail of minimum essential medium (MEM) α containing 10% fetal bovine serum (FBS), 1.0 μM dexamethasone, 0.5 mM 3-isobutyl-1-methylxanthine, and 0.01 mg/ml insulin. Adipogenesis was confirmed by staining with Oil Red O after 14 days. For osteoblast differentiation, MSCs were incubated in MEM α containing 10% FBS, 100 mM dexamethasone, 10 mM sodium glycerophosphate, and 50 ng/ml vitamin C. Osteogenesis was confirmed by Alizarin Red staining after 14 days.

This study was approved by the Ethics Committee of the Jiujiang University Subsidiary Hospital and followed the Declaration of Helsinki. The informed consent of all subjects was kindly obtained from bone marrow donors prior to the study.

### Transposition reaction and PCR amplification

Transposition and PCR were performed as previously described [16]. Briefly, after adipogenic and osteogenic induction of MSCs, cells from each group were harvested and digested into single-cell suspensions. For the transposition reaction, 5 × 10^4^ cells from each group were centrifuged for 5 min at 500 × g at 4 °C and the supernatant was discarded. After washing with cold phosphate-buffered saline (PBS), the cells were resuspended by gentle pipetting in 50 μl of chilled lysis buffer (10 mM Tris–HCl, pH 7.4, 10 mM NaCl, 3 mM MgCl2, 0.1% IGEPAL CA-630) and immediately centrifuged for 10 min as above. The precipitate was resuspended in the transposition reaction mixture from the Nextera kit (Illumina, San Diego, CA, USA) consisting of 25 µl TD (2 × reaction buffer from the kit), 2.5 µl TDE1 (Nextera Tn5 Transposase from the kit), and 22.5 µl nuclease-free H_2_O. The mixture was then incubated at 37 °C for 30 min. The reaction products were then purified using a Qiagen MinElute PCR Purification Kit (Qiagen, Hilden, Germany) and eluted in 10 μl elution buffer. Before amplifying the transposed DNA fragments, the following reagents were mixed: 10 µl transposed DNA, 10 µl nuclease-free H_2_O, 2.5 µl of 25 µM PCR Primer 1, 2.5 µl of 25 µM Barcoded PCR Primer 2, and 25 µl NEBNext High-Fidelity 2 × PCR Master Mix in a 0.2 ml PCR tube. The thermal cycle was as follows: 72 °C for 5 min, 1 cycle; 98 °C for 30 s; 98 °C for 10 s, 5 cycles; 63 °C for 30 s; 2 °C for 2 min. The amplified products were purified by the Qiagen MinElute PCR Purification Kit.

### Sequencing and data analysis

ATAC-sequencing was performed by Jiayin Biotechnology Ltd (Shanghai, China) on an Illumina HiSeq platform (Illumina, San Diego, CA, USA) according to commercially available protocols. Fast QC software was used for the quality control of sequencing data. BWA software compared the clean data to the reference genome hg38_genecode [17]. The BAM file obtained after comparison and analysis was used as the input file, and the peaks were called using MACS2 software, with a filtering threshold of Q < 0.05 [18]. Each peak region extended 200 bp from the 5’ end to the 3’ end to extract the DNA sequence. The motif was predicted using Homer software and the predicted motif was matched with the motif data in the database (Homer and Jaspar) and known motifs and corresponding transcription factors were screened out. Deeptools software was used to analyze the signal distribution near the gene. Gene Ontology (GO) was used to elucidate the biological processes (BPs) of genes associated with the accessible chromatin regions. The Kyoto Encyclopedia of Genes and Genomes (KEGG) database was used to analyze enriched pathways of the genes adjacent to the peaks.

### RNA-seq

To relate chromatin accessibility to mRNA expression, we performed RNA-seq on the same batch of cells used for ATAC-seq. Total RNA was extracted from individual samples using the TRIzol reagent (Invitrogen). The RNA quality was confirmed using an by Agilent 2200 (Agilent Technologies, Santa Clara, CA, USA) and stored at − 80 °C. RNA with a RIN (RNA integrity number) > 7.0 was considered acceptable for cDNA library construction. cDNA libraries were constructed for each RNA sample using the TruSeq Stranded mRNA Library Prep Kit (Illumina) according to the manufacturer’s instructions. Briefly, the protocol consisted of the following steps: Poly-A containing mRNA was purified from 1 µg total RNA using oligo (dT) magnetic beads and cleaved into 200–600 bp fragments with divalent cations at 85 °C for 6 min. The cleaved RNA fragments were used for first- and second-strand complementary DNA (cDNA) synthesis. A dUTP mix was used for second-strand cDNA synthesis, which allows the removal of the second strand. The cDNA fragments were end-repaired, A-tailed, and ligated with indexed adapters. The ligated cDNA products were purified and treated with uracil DNA glycosylase to remove the second-strand cDNA. Purified first-strand cDNA was enriched by PCR to create the cDNA libraries. The quality of the libraries was monitored with the Agilent 2200 and sequenced using a NovaSeq 6000 platform (Illumina) on a 150 bp paired-end run.

### Combination of the ATAC-seq and RNA-seq data

To integrate the ATAC-seq and RNA-seq data, the overlap between the down-regulated differentially expressed genes identified by RNA-seq and the related genes of the open chromatin region showing reduced ATAC-seq signals were determined. A similar analysis was performed for the upregulated genes identified by RNA-seq and chromatin genes with increased ATAC-seq signals.

### Real-time PCR verification

The RNA-seq data were verified using qRT-PCR. Specifically, the sequencing data of 12 genes identified as being closely associated with adipogenesis or osteogenesis were analyzed using a 7500 Real-Time PCR System (ABI, Foster, CA, USA). The primers used are listed in Additional file 1: Table S1. First-strand cDNA synthesis was performed using ReverTra Ace® qPCR RT Kit (Toyobo, Osaka, Japan), and PCR used the SYBR Green Realtime PCR Master Mix (Toyobo) at 94 °C for 5 min, 58 °C for 34 s, and a final extension at 72 °C with 30 cycles. β-actin was used as the internal control.

## Results

### Landscape of chromatin accessibility

**Fig. 1:**
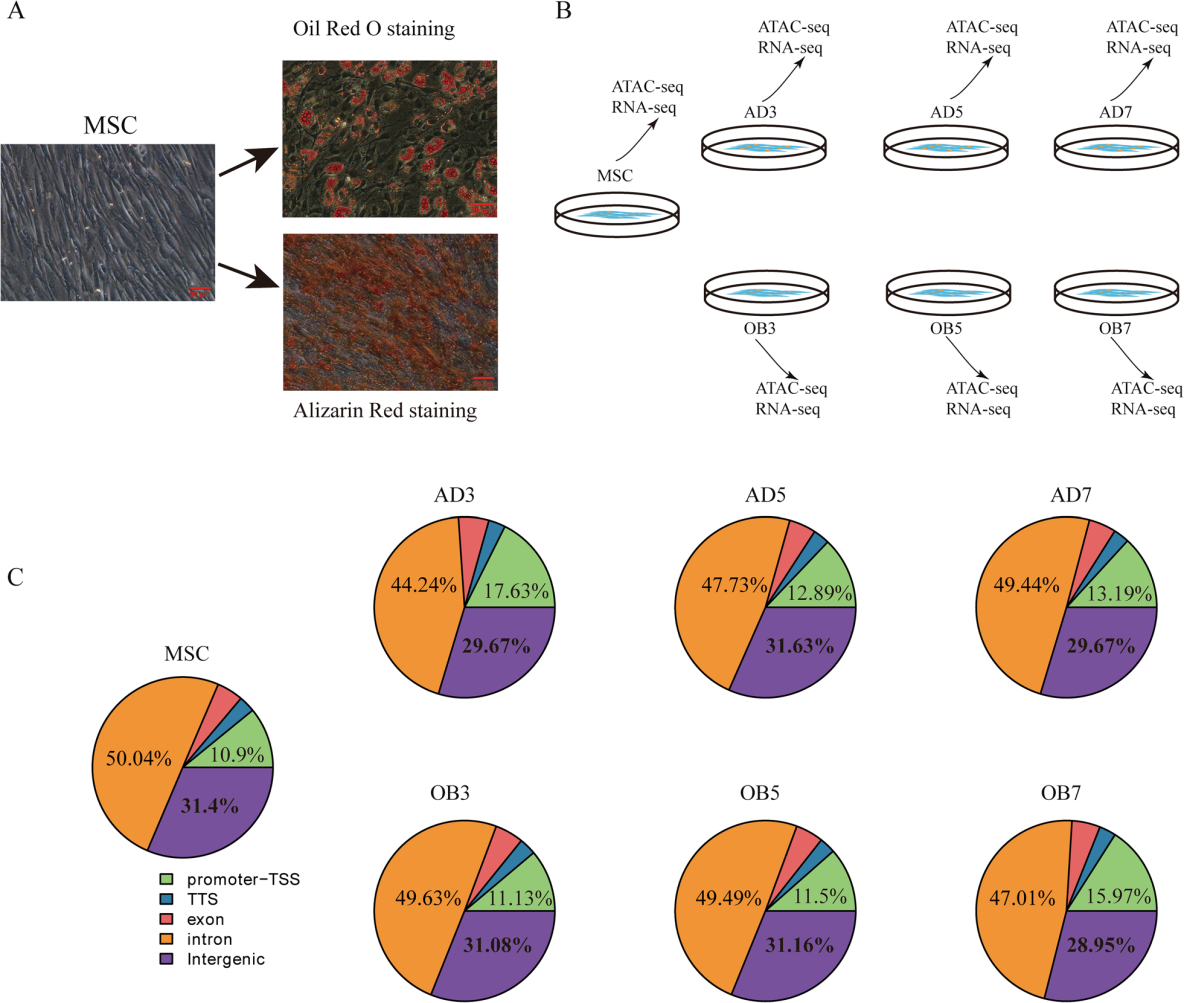
ATAC sequencing analysis of adipogenic and osteogenic differentiation of MSCs. **A** Adipogenic and osteogenic differentiation of MSCs demonstrated by Oil Red O and Alizarin Red staining, respectively, on day 14; **B** Cell group pattern diagram; **C** Peak distributions in the functional regions of the genome shown by statistical analysis

Sixth-generation primary hMSCs were stimulated by adipogenic or osteogenic inducers, and the differentiated morphology was confirmed by Oil Red O staining and Alizarin Red staining, respectively (Fig. 1a). Based on the successful differentiation, we harvested cells at specified times during differentiation for ATAC-seq and RNA-seq analysis (Fig. 1b).

We first conducted ATAC-seq. Data quality checking showed that the rate of effective reads in each group was higher than 90% (Table 1), indicating the reliability of the sequencing. The peak numbers in each group calculated by MACS2 software are listed in Table 1. Notably, the number of accessible chromatin regions decreased significantly in the adipogenic AD3 group, and gradually recovered in the AD5 and AD7 groups. In contrast, there were minimal changes in the numbers of accessible chromatin regions in the osteogenic OB3 and OB5 groups, although a significant reduction was seen in the OB7 group. Analysis of the read distributions using Deeptools showed that the majority of the read signals in each group were concentrated near the transcriptional start sites (TSS), and were also concentrated near the centers of peaks (Additional file 2: Fig. S1A and B), indicating the reliability of the sequencing. The accessible peaks in each group were widely distributed throughout the genome (Fig. 1c). Specifically, the proportions of peaks in the intronic and intergenic regions were relatively high (approximately 80%), while the percentages of peaks in the promoter regions fluctuated between 10 and 20% in all groups. In adipogenic differentiation, the highest proportion of accessible chromatin regions located in the promoter regions was seen in the AD3 group (17.63%), while the number of read signals was the lowest (68,327). Conversely, in the osteoblast differentiation groups, the highest percentage of accessible chromatin regions located in the promoters was in the OB7 group (15.98%), which also showed the lowest peak number (68,216). As intergenic regions account for a large proportion of the genome, more peaks are detected in these regions; however, as these are non-coding regions, these peaks generally do not represent actual regulatory factor-binding sites.

**Table 1:** Summary of ATAC-seq, read mapping, motif, and peak calling results

	MSC	AD3	AD5	AD7	OB3	OB5	OB7
Mapped reads	144,722,564	142,125,644	138,602,489	199,777,605	149,649,854	146,697,518	144,753,928
Mapped rate	99%	98.1%	98.5%	96.8%	96.7%	91.8%	97.5%
Peak numbers	110,369	68,327	99,362	77,712	113,785	110,267	68,216
Motif binding	270,998	150,410	220,839	152,472	295,624	275,090	98,411
Motif types	577	555	562	533	570	560	522
Gene numbers*	19,264	20,193	19,227	19,936	19,348	19,023	18,825

*RNA-seq results

### Motif analysis

We used HOMER to identify transcription factor-binding motifs enriched in the accessible chromatin regions. The total numbers and types of motifs showed minimal variation between the different groups (Table 1). The top 10 major motifs identified in each group are listed in Additional file 3: Table S2, with most of them, such as fra1, fra2, ATF3, junB, BATF, and AP-1, belonging to the basic leucine zipper (bZIP) transcription factor family. We next examined alterations in these motifs during the differentiation process. Using MSC as the control group, the major changes in the motifs in the different groups are shown in Table 2. Specifically, during adipogenic differentiation, the top motifs identified in the AD3 and AD5 groups remained similar, specifically, motifs belonging to the CEBP, NFIL3, and HLF families, while in AD7 group, the most common motifs belonged to the TEAD family. Interestingly, the number of RUNX family motifs began to increase in the AD5 group. On the other hand, during osteogenic differentiation, the transcription factor families showing the greatest increase were TEAD and RUNX in the OB3 and OB5 groups, changing to CEBP, NFIL3, and HLF in the OB7 group. Interestingly, no clear pattern was discernable for the decreased motifs.

**Table 2:** The 10 top-ranking motifs that changed with differentiation in each group

Rank	AD3 versus MSC		AD5 versus MSC		AD7 versus MSC		OB3 versus MSC		OB5 versus MSC		OB7 versus MSC	
	Up	Down	Up	Down	Up	Down	Up	Down	Up	Down	Up	Down
1	CEBP	Atf3	CEBP	Fra1	TEAD3	Fra1	TEAD3	Fra1	RUNX1	Atf3	CEBP	Fra1
2	NFIL3	Fra1	CEBP:AP1	Atf3	TEAD1	Atf3	TEAD1	Atf3	TEAD1	Fra1	EBF2	Atf3
3	HLF	BATF	HLF	BATF	TEAD4	BATF	TEAD4	AP-1	TEAD3	AP-1	NFIL3	BATF
4	EBF2	AP-1	NFIL3	Fra2	TEAD	AP-1	RUNX	BATF	TEAD4	BATF	HLF	Fra2
5	CEBP:AP1	JunB	RUNX1	AP-1	CEBP	Fra2	RUNX1	JunB	RUNX2	JunB	CEBP:AP1	JunB
6	NF1	Fra2	EBF2	JunB	RUNX2	JunB	RUNX2	Fra2	RUNX-AML	Fra2	NF1	AP-1
7	Atf4	Fosl2	RUNX2	Fosl2	RUNX1	Fosl2	CEBP	Fosl2	TEAD2	Fosl2	EBF1	Fosl2
8	EBF1	Jun-AP1	RUNX-AML	Jun-AP1	NF1	Jun-AP1	TEAD2	Jun-AP1	CEBP	Jun-AP1	Atf4	Jun-AP1
9	GRE	Bach2	Atf4	Bach2	RUNX-AML	Bach2	RUNX-AML	Bach2	NF1	Bach2	GRE	Bach2
10	EBF	MafK	NF1	MafK	TEAD2	MafK	CEBP:AP1	Bach1	CEBP:AP1	Bach1	ARE	Bach1

Since PPARγ and RUNX2 are considered to be key regulatory transcription factors in adipogenic and osteogenic differentiation, respectively, we specifically tracked their dynamic enrichment. As expected, the relative activity of the PPARγ motif was increased in the AD3 and AD5 groups but diminished in the AD7 group. Similarly, the relative activity of RUNX2 was enhanced in the OB3 and OB5 groups but decreased in the OB7 group (Additional file 4: Fig. S2).

### Functional enrichment analysis of genes identified by ATAC-seq

**Fig. 2:**
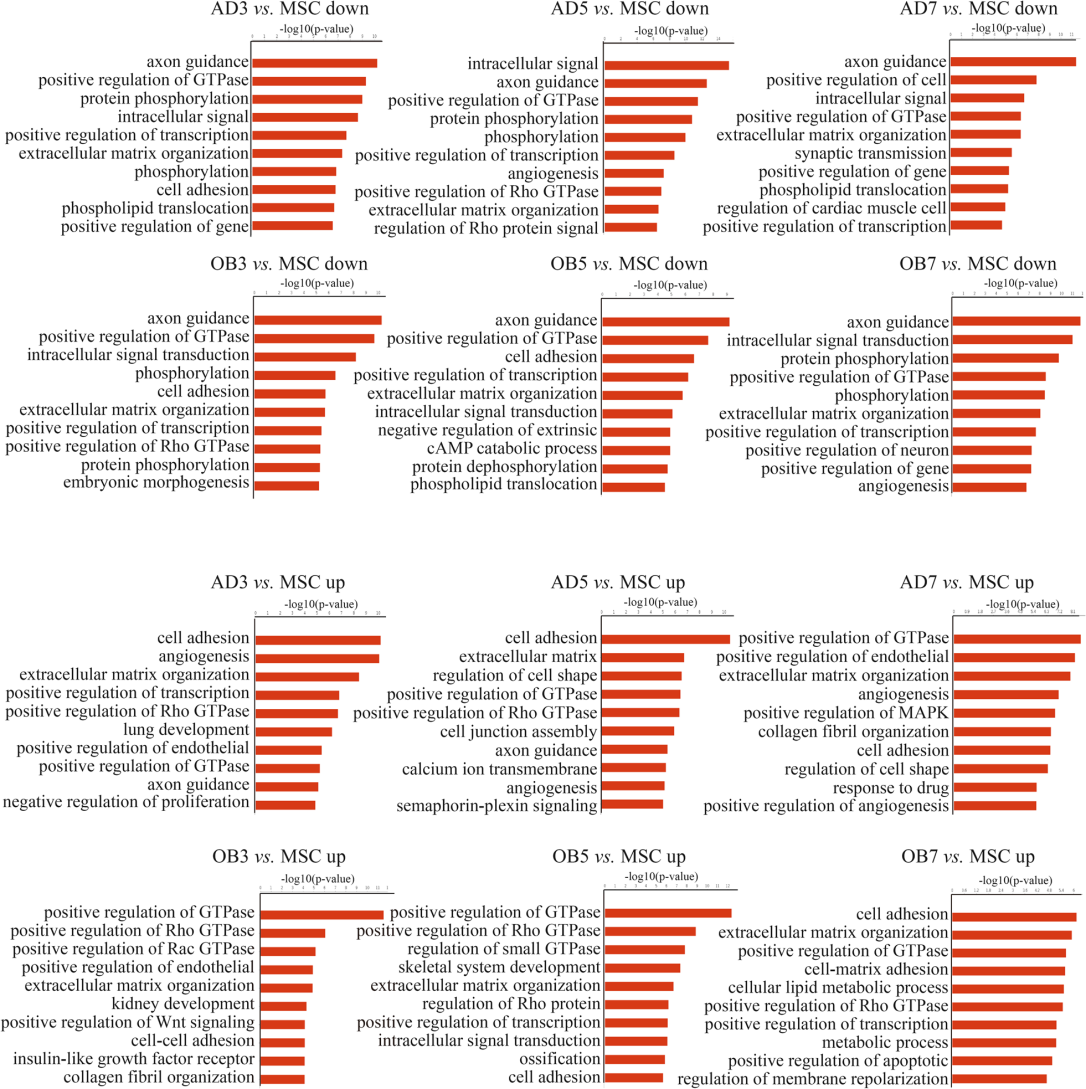
Gene ontology analysis of genes associated with differences in accessible chromatin regions

The biological process (BP) analysis in GO showed that genes associated with accessible chromatin regions were most enriched in the “phosphorylation”, “metabolic process”, and “protein transport” categories. This was found in all groups and indicates the importance of maintaining basic cellular functions (Additional file 5: Table S3). The top BP terms from the GO analysis are shown in Fig. 2. In adipogenic differentiation, four BPs including “cell adhesion”, “angiogenesis”, “extracellular matrix organization”, and “positive regulation of GTPase activity” were consistently up-regulated in each group. In comparison, during osteogenic differentiation, three BPs including “positive regulation of GTPase activity”, “positive regulation of Rho GTPase activity”, and “extracellular matrix organization” were consistently up-regulated in each group. Interestingly, whether during adipogenic or osteogenic differentiation, the function of the top-ranking up-regulated genes on the seventh day was significantly different from that on the third and fifth days.

**Fig. 3:**
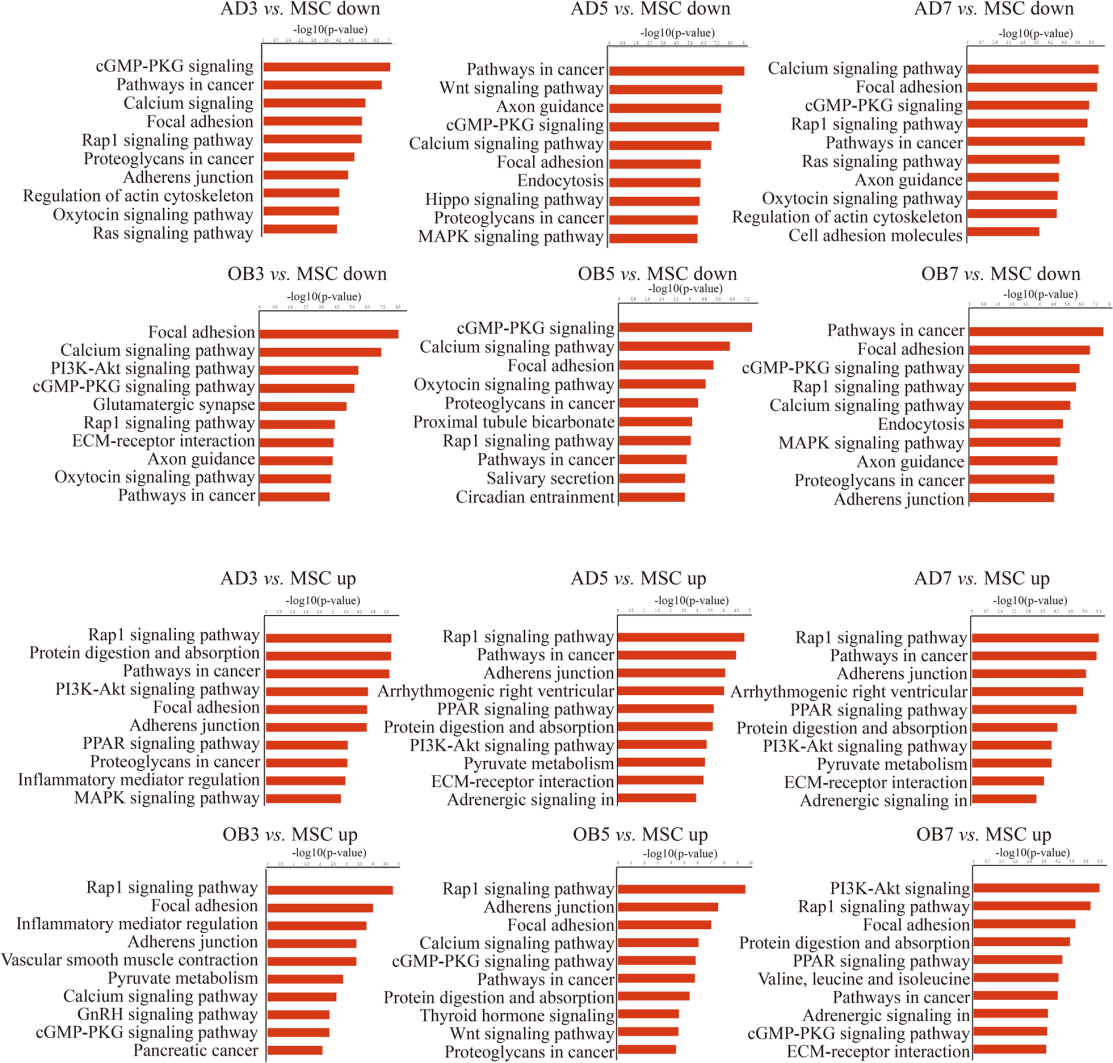
KEGG pathway analysis of genes associated with differences in accessible chromatin regions

The genes associated with accessible chromatin regions were then subjected to KEGG pathway analysis. The top 10 pathway terms in each group are listed Additional file 6: Table S4. The major pathways identified in each group included “cancer”, “regulation of actin cytoskeleton”, “proteoglycans in cancer”, “focal adhesion”, and “insulin signaling pathway”. The top 10 pathways that showed changes in the differentiated cells and MSCs are seen in Fig. 3. Five pathways were consistently up-regulated in adipogenic differentiation, including “Rap1 signaling pathway”, “pathways in cancer”, “adherens junction”, “protein digestion and absorption”, and “PI3K-Akt signaling pathway”. On the other hand, three pathways were consistently up-regulated during osteogenic differentiation, including “Rap1 signaling pathway”, “focal adhesion”, and “cGMP-PKG signaling pathway”.

### RNA-seq results

**Fig. 4:**
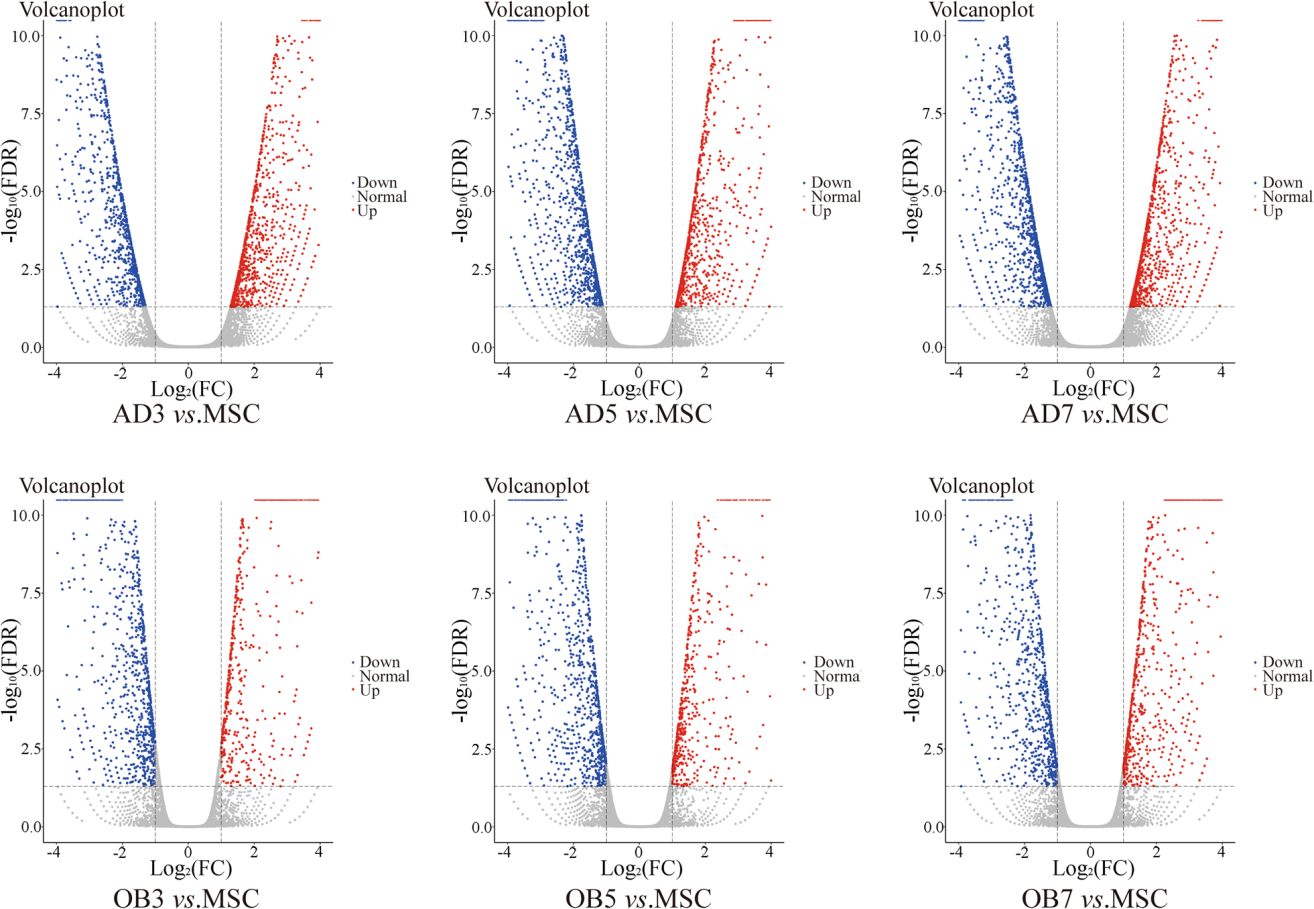
Volcano plots of differentially expressed genes in each group

RNA-seq was performed on the same batches of cells as ATAC-seq, and no significant differences were observed in the numbers of genes identified in each group (Table 1). Using log2fc > 1 or < − 1, FDR < 0.05 as the screening criteria, genes showing significantly different expression were screened out; these are shown in Table 3 and Fig. 4. We observed that there was more variation in the numbers of genes during adipogenesis than during osteogenesis. The differentially expressed genes identified by RNA-seq were then analyzed by GO and KEGG, and histograms of the enriched categories are shown in Additional file 7: Fig. S3 and Additional file 8: Fig. S4. Unexpectedly, the enriched BPs and pathways seen in RNA-seq were quite different from those seen in ATAC-seq. For example, the “fatty acid metabolism” and “adipocytokine signaling pathway” pathways were significantly up-regulated in RNA-seq but not in ATAC-seq during adipogenic differentiation. Similarly, “Rap1 signaling pathway” and “focal adhesion” were significantly up-regulated in ATAC-seq but not in RNA-seq during osteogenic differentiation.

**Table 3:** Comparison of the numbers of differentially expressed genes between groups during the early stage of osteogenic/adipogenic differentiation

Comparison	Down gene num.*	Up gene num.*
AD3 versus MSC	1305	1454
AD5 versus MSC	1723	1308
AD7 versus MSC	1763	1581
OB3 versus MSC	1162	720
OB5 versus MSC	1214	698
OB7 versus MSC	1087	1016

*The screening criteria for differentially expressed genes were log2fc > 1 or < − 1, FDR < 0.05

*Down* downregulated, *Up* upregulated

### Combined ATAC-seq and RNA-seq analysis

**Fig. 5:**
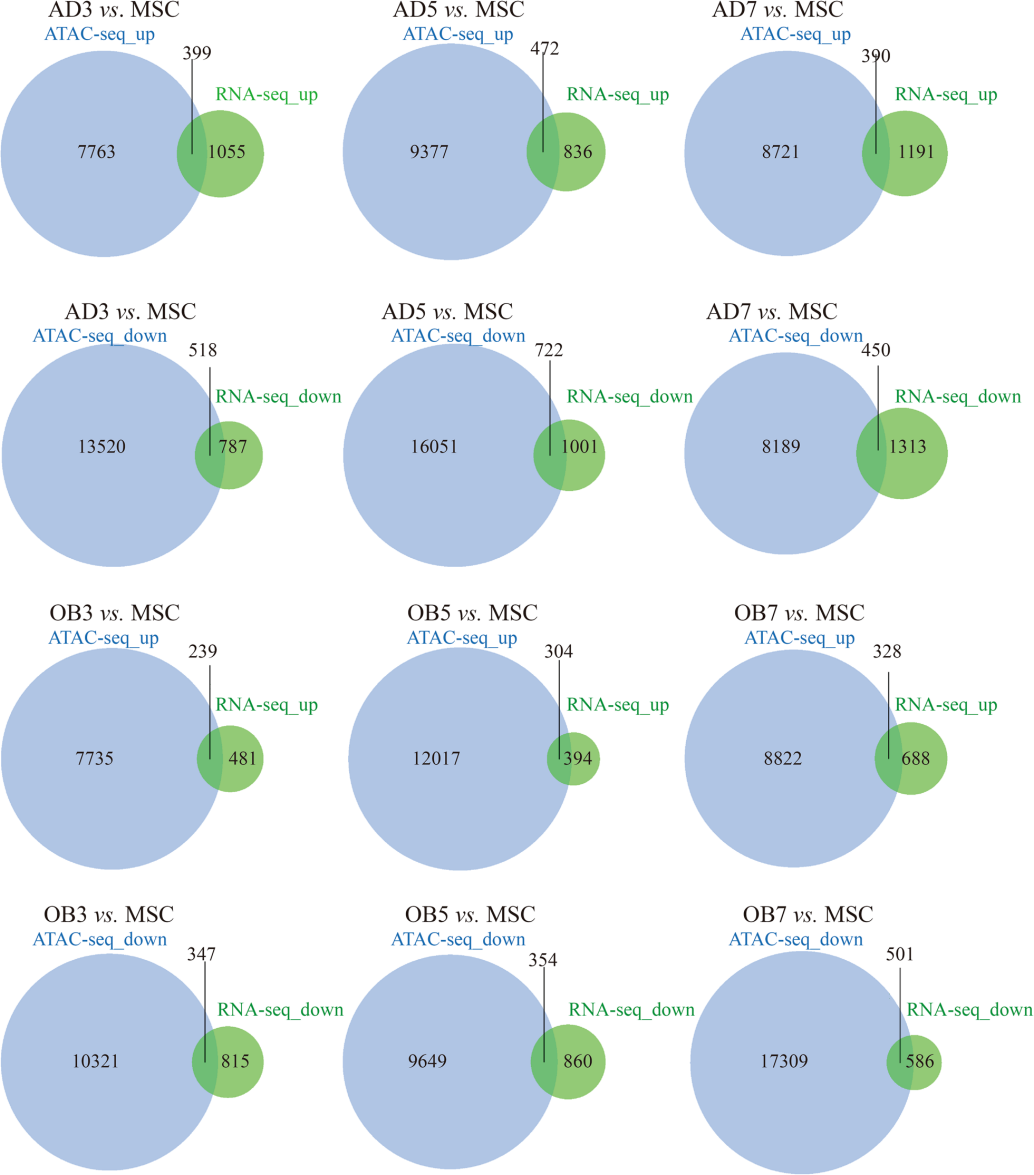
Numbers of overlapping genes between the ATAC-seq and RNA-seq data for the different groups

The number of overlapping genes between ATAC-seq and RNA-seq for the different groups are shown in Fig. 5. In terms of proportion, overlapping genes accounted for only a small proportion of the genes identified by ATAC-seq but a higher proportion of the total genes from RNA-seq.

The top 10 BP terms from GO analysis of the overlapping genes are shown in Additional file 9: Fig. S5. During adipogenic differentiation, “small molecule metabolic process” and “lipid metabolic process” were significantly up-regulated in the AD3 and AD5 groups. In osteogenic differentiation, in contrast, considerable variation was observed in the top 10 enriched BPs, with “extracellular matrix organization” being the only BP which was significantly up-regulated in each group.

The overlapping genes were also subjected to KEGG pathway analysis. The top 10 BP terms in each group are shown in Additional file 10: Fig. 6. In adipogenic differentiation, “PPAR signaling pathway” and “fatty acid metabolism” were significantly up-regulated in the AD3 and AD5 groups while, during osteogenic differentiation, “TGF-β signaling pathway” and “focal adhesion” were significantly up-regulated in the AD3 and AD5 groups.

**Fig. 6:**
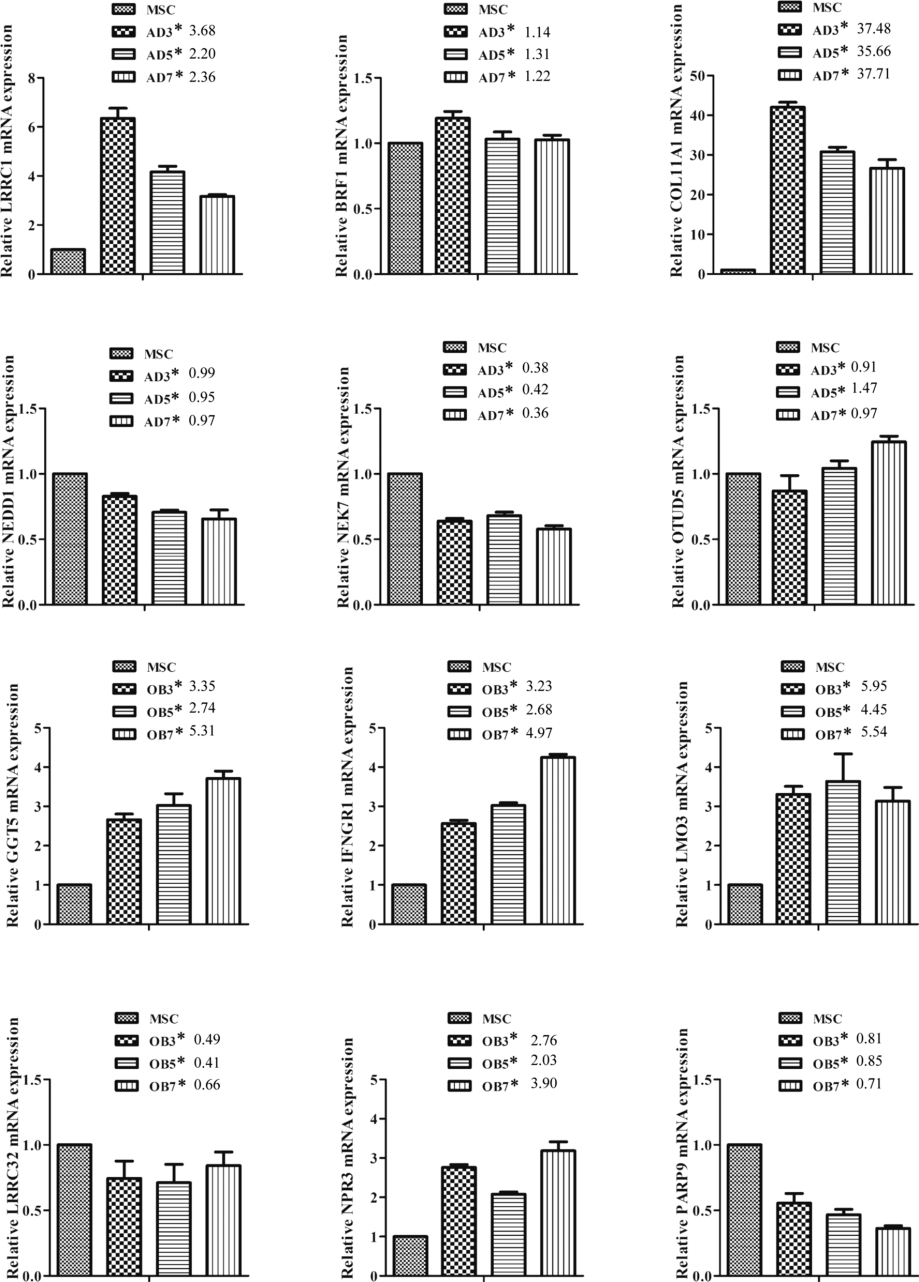
RT-PCR verification of 12 genes identified by RNA-seq. *Fold changes compared with MSC from RNA-seq data

### PCR verification

We randomly selected 12 genes from the overlapped genes for qRT-PCR verification which showed that the expression trend of these representative genes were almost consistent with the RNA-seq data, indicating the reliability of the sequencing results (Fig. 6).

## Discussion

Differentiation of MSCs into adipocytes or osteoblasts is a complex process. During each process, the MSCs are required to undergo lineage commitment stage and cellular maturation, accompanied by substantial alterations in gene expression [19]. However, the mechanisms of transcriptional activation of key genes are not entirely clear. Therefore, we have attempted to describe the chromatin accessibility in combination with gene transcription at the early stages after induction for an in-depth understanding of the regulatory mechanisms involved in MSC differentiation.

ATAC-seq technology differs from RNA-seq in that it can provide information on accessible chromatin regions at a genome-wide level and at specific times, allowing the effective detection of patterns in transcriptional activation under specific conditions. ATAC-seq has been applied to the investigation of numerous complex diseases, including cutaneous T cell lymphoma [20] and diabetes [21], amongst others. It has also been used in MSCs to identify cellular diversity from different tissue origins [22] and used to study the chromatin architecture in epidermal MSCs [23]. In contrast to these earlier studies, we focused on changes in chromosome accessibility during the directional differentiation of MSCs. The sequencing data showed that cultured MSCs have more than a hundred thousand accessible chromatin regions, mainly concentrated near the transcription starting point, and consistent with previously published ATAC-seq data on MSCs [22]. However, due to the differences in growth environments in vitro and in vivo, our results cannot yet predict the accessible chromatin conditions in MSCs in vivo.

We observed that chromatin accessibility changes dynamically during MSC early-stage differentiation. This is the result not only of external stimulation but also caused by alternations in gene expression. Interestingly, the numbers of expressed genes identified by RNA-seq did not change significantly between time points, while significant fluctuation was seen in the ATAC-seq data; a possible explanation is that chromatin accessibility is a more accurate means for identifying cell types than transcriptome analysis [22]. Our results show that overlapping genes between the ATAC-seq and RNA-seq data only accounts for a small part of the enriched genes identified by ATAC-seq, indicating that changes in chromatin accessibility do not correspond perfectly with changes in gene expression. These complex cytological functions require further exploration.

A useful feature of ATAC-seq is that it reveals transcription factor-binding motifs under physiological conditions [16]. We observed that the most frequently identified motifs identified in each differentiation group were members of the bZIP family, suggesting that bZIP family members play important roles in maintaining basic cellular functions [24]. We focused on differentially enriched motifs at different time-points during the differentiation process. During adipogenic and osteogenic differentiation, the CREB motif was the most significantly up-regulated motif in the AD3 and AD5 groups, highlighting its importance during adipogenesis [25]. In addition to CREB, NFIL3 and HLF were also significantly up-regulated in the AD3 and AD5 groups. The bZIP transcription factor NFIL3, also known as E4BP4, regulates a variety of physiological processes ranging from viability to the circadian rhythm [26]. Notably, NFIL3 was found to be significantly up-regulated during adipogenesis and to mediate glucocorticoid-regulated adipogenesis [27]. In addition, HLF is a member of the bZIP transcription factor family [28] and is believed to participate in the physiological regulation of cellular lipid levels [29]. The precise roles of NFIL3 and HLF in adipogenesis are worthy of further study. During osteogenesis, the TEAD and RUNX families were observed to be the most significant up-regulated motifs in the first five days. Compared with the RUNX family of transcription factors, the role of the TEAD family in osteogenic differentiation has been less studied. TEAD family members contain a strongly conserved DNA-binding domain, the TEA domain [30], and TEAD2 was identified as a novel regulator of osteogenesis [31], indicating the potential role of the TEAD family in the regulation of the osteogenic process. We noticed that the up-regulated motifs enriched on the seventh day were quite different compared with those observed on the fifth and third days, during both adipogenic and osteogenic differentiation, indicating a significant change in the cell function on the seventh day of MSC differentiation. Interestingly, CREB, NFIL3, and HLF, all of which were up-regulated in the first five days of adipogenic differentiation, were only up-regulated in the OB7 group during osteogenesis; correspondingly, TEADs and RUNX2, which were up-regulated in the first five days of the osteogenic process, were only activated in the AD7 group in adipogenesis, suggesting a balanced regulation between the processes of adipogenic and osteogenic differentiation [32].

Functional enrichment analysis is a method to identify classes of genes, which helps to better understand the potential biological processes in gene sets [33]. We observed that the enriched genes from both the ATAC-seq and RNA-seq data showed clear differences in terms of BP and KEGG pathway analyses, demonstrating the difference in application scope between ATAC-seq and RNA-seq. Nevertheless, a small number of overlapping genes between the ATAC-seq and RNA-seq data shared common biological functions. For example, during adipogenic differentiation, both ATAC-seq and RNA-seq showed increased enrichment of the PPAR pathway and fatty acid metabolism, both of which are related to adipogenesis. In parallel, up-regulation of the TGF-β and focal adhesion pathways demonstrate synergism between chromatin structure and gene expression during osteogenic differentiation. In addition, there were also some BPs and KEGG pathways that are seemingly unrelated to MSCs differentiation, according to previous studies, such as the axon guidance and neurotrophin signaling pathways, and these warrant further study.

In summary, we used ATAC-seq to describe and analyze chromosomal accessibility and gene expression in MSCs during adipogenic/osteogenic differentiation. This work will help to reveal the network of gene loci and transcription factors in cell differentiation and provide more evidence for understanding the changes of epigenetic programming in MSC cells.

## Supplementary Information

**Additional file 1:** The primer sequences were used for real time PCR.

**Additional file 2:** Read signals in each group were concentrated near the transcriptional start sites and centers of peaks.

**Additional file 3:** The top 10 major motifs identified in each differentiated group.

**Additional file 4:** Relative activity of PPARγ and RUNX2 motif in adipogenic and osteogenic differentiation.

**Additional file 5:** GO analysis of genes associated with accessible chromatin region.

**Additional file 6:** KEGG pathway analysis of genes associated with accessible chromatin regions.

**Additional file 7:** GO analysis of differentially expressed genes identified by RNA-seq.

**Additional file 8:** KEGG pathway analysis of differentially expressed genes identified by RNA-seq.

**Additional file 9:** GO analysis of overlapped genes from ATAC-seq and RNA-seq data.

**Additional file 10:** KEGG pathway analysis of overlapped genes from ATAC-seq and RNA-seq data.

## Data Availability

Sequencing data of RNA-Seq in this study can be found with accession number of GSE174794 (https://www.ncbi.nlm.nih.gov/geo/query/acc.cgi?acc=GSE174794) in the GEO database at NCBI. Sequencing data of ATAC-Seq are available from the corresponding author on reasonable request.
