# Supplementary material for: Alterations in chromatin accessibility during osteoblast and adipocyte differentiation in human mesenchymal stem cells
Source: BMC Med Genomics. 2022 Jan 31;15:17. doi: 10.1186/s12920-022-01168-1 (PMC8802426; doi:10.1186/s12920-022-01168-1)
Supplement: Supplementary file 1 — Additional file 1: The primer sequences were used for real time PCR. [file 12920_2022_1168_MOESM1_ESM.docx]

Supplementary Table 1. The primer seqnences were used for real time PCR

| Gene | Forward primer: 5’-3’ | Reverse primer 5’-3’ |
| --- | --- | --- |
| LRRC1 | CAGACTAACTCGGATACCTGCAG | CTGGTTGTCAGATAGCCACAGAG |
| BRF1 | AGAACCACGAGGTGTCCATGAC | CCACACTGATGACCTCCTTCAC |
| COL11A1 | ATGGACCAGCAGGATTACGTGG | TGTACCTGCTGACCCACGTTCT |
| NEDD1 | GCTCTTTGTAACCATAGGCTTGG | CCAAAGTGGCTCCATCAGGCAT |
| NEK7 | AGAACAGTGTGACTACCCACCTC | CATAGGTGACGTCTGGTCGCTT |
| OTUD5 | CAGGCTACAACAGTGAGGACGA | GAAGCCCTTCTTGTCTCGTAGG |
| GGT5 | CAGATGCTGGTGGAGGACATTG | GTGGTGAGTACAGGGTATAGTCC |
| IFNGR1 | AGTGCTTAGCCTGGTATTCATCTG | GGCTGGTATGACGTGATGAGTG |
| LMO3 | ATGGTGATGCGTGCCAAGGACA | AACCTTCCTCGTAGTCCGTCTG |
| LRRC32 | GCATAGCAACGTGCTGATGGAC | GATGCTGTTGCAGCTCAGGTCT |
| NPR3 | CAGTGGAGACTACGCCTTCTTC | TGACTGTCTGGAGGGACGAGTA |
| PARP9 | GGCAAAGAGGTCCAAGATGCTG | GCCTCACACATCTCTTCCACGT |
| Beta actin | CACCATTGGCAATGAGCGGTTC | AGGTCTTTGCGGATGTCCACGT |
