# Supplementary figures and images for: Alterations in chromatin accessibility during osteoblast and adipocyte differentiation in human mesenchymal stem cells
Source: BMC Med Genomics. 2022 Jan 31;15:17. doi: 10.1186/s12920-022-01168-1 (PMC8802426; doi:10.1186/s12920-022-01168-1)

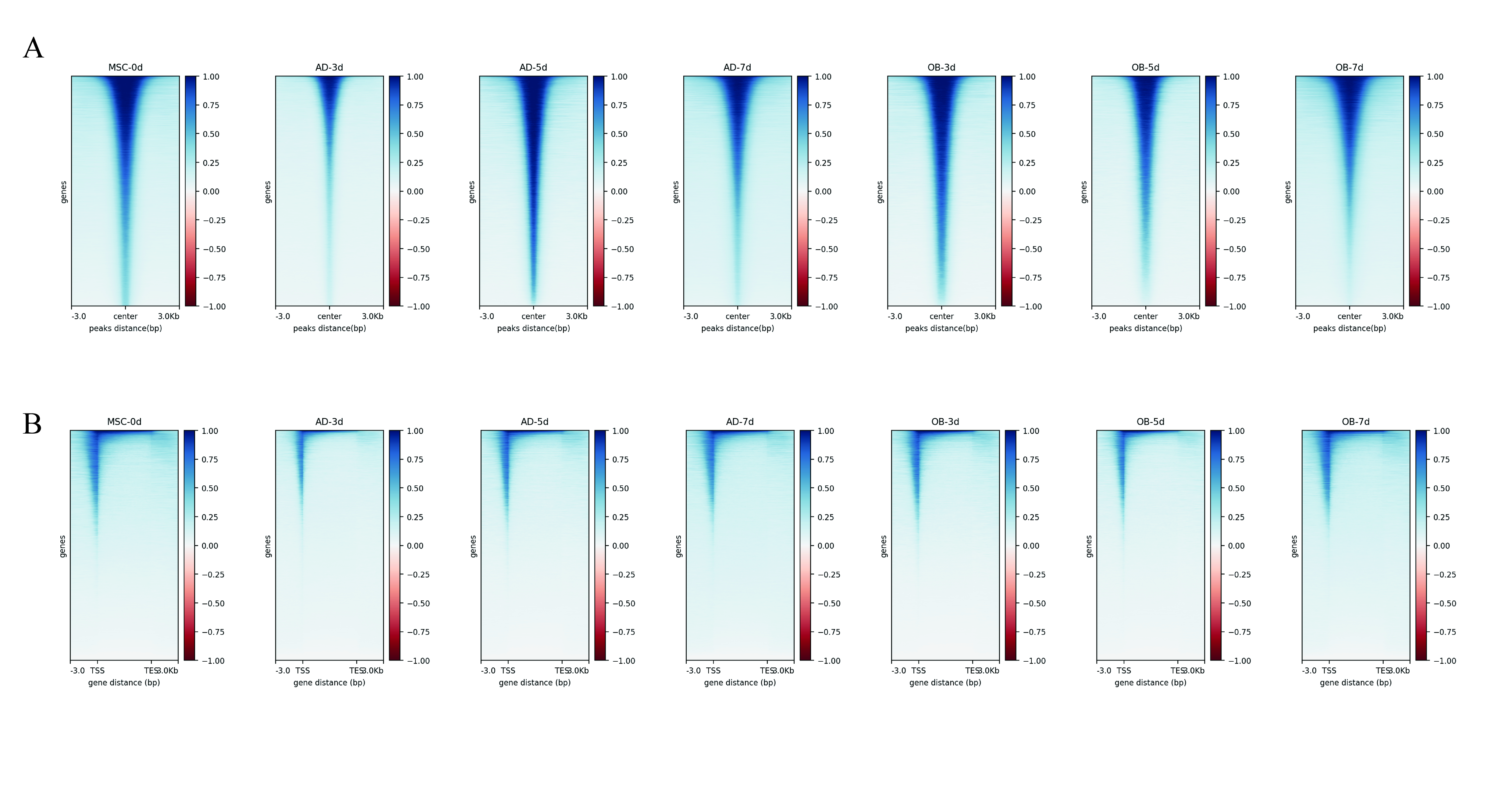

Supplement: Supplementary file 2 — Additional file 2: Read signals in each group were concentrated near the transcriptional start sites and centers of peaks. [file 12920_2022_1168_MOESM2_ESM.tif]

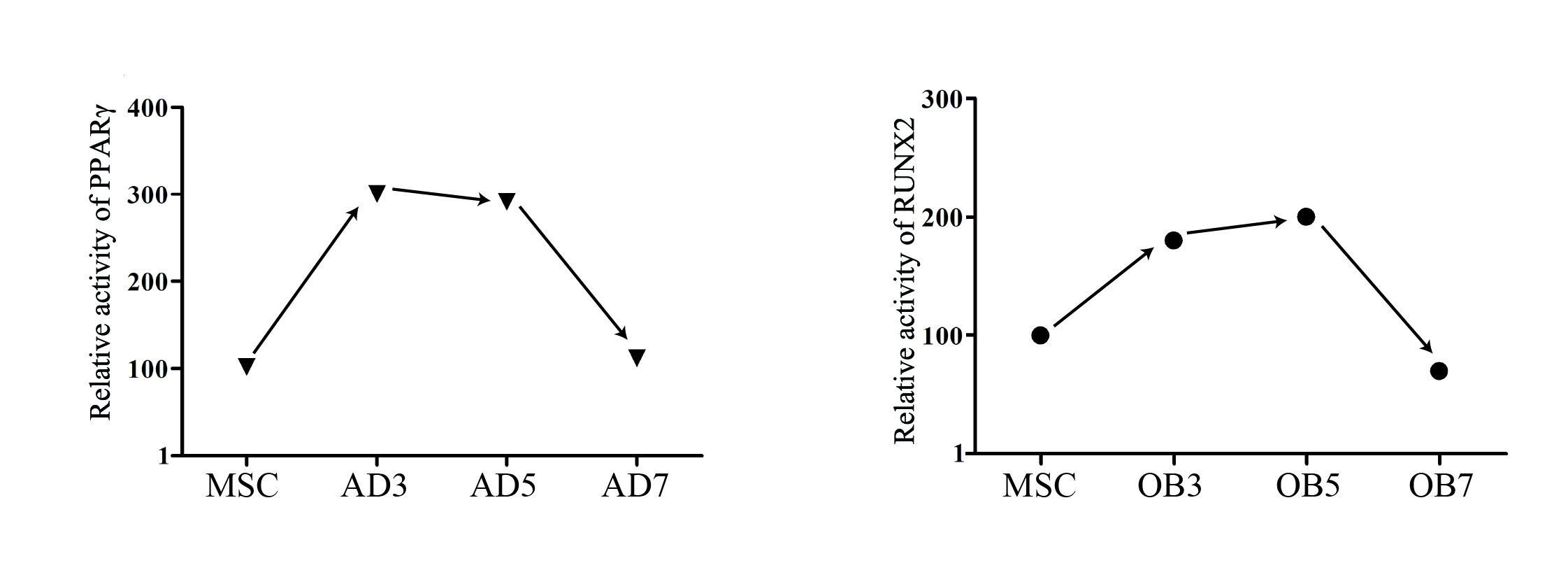

Supplement: Supplementary file 4 — Additional file 4: Relative activity of PPARγ and RUNX2 motif in adipogenic and osteogenic differentiation. [file 12920_2022_1168_MOESM4_ESM.tif]

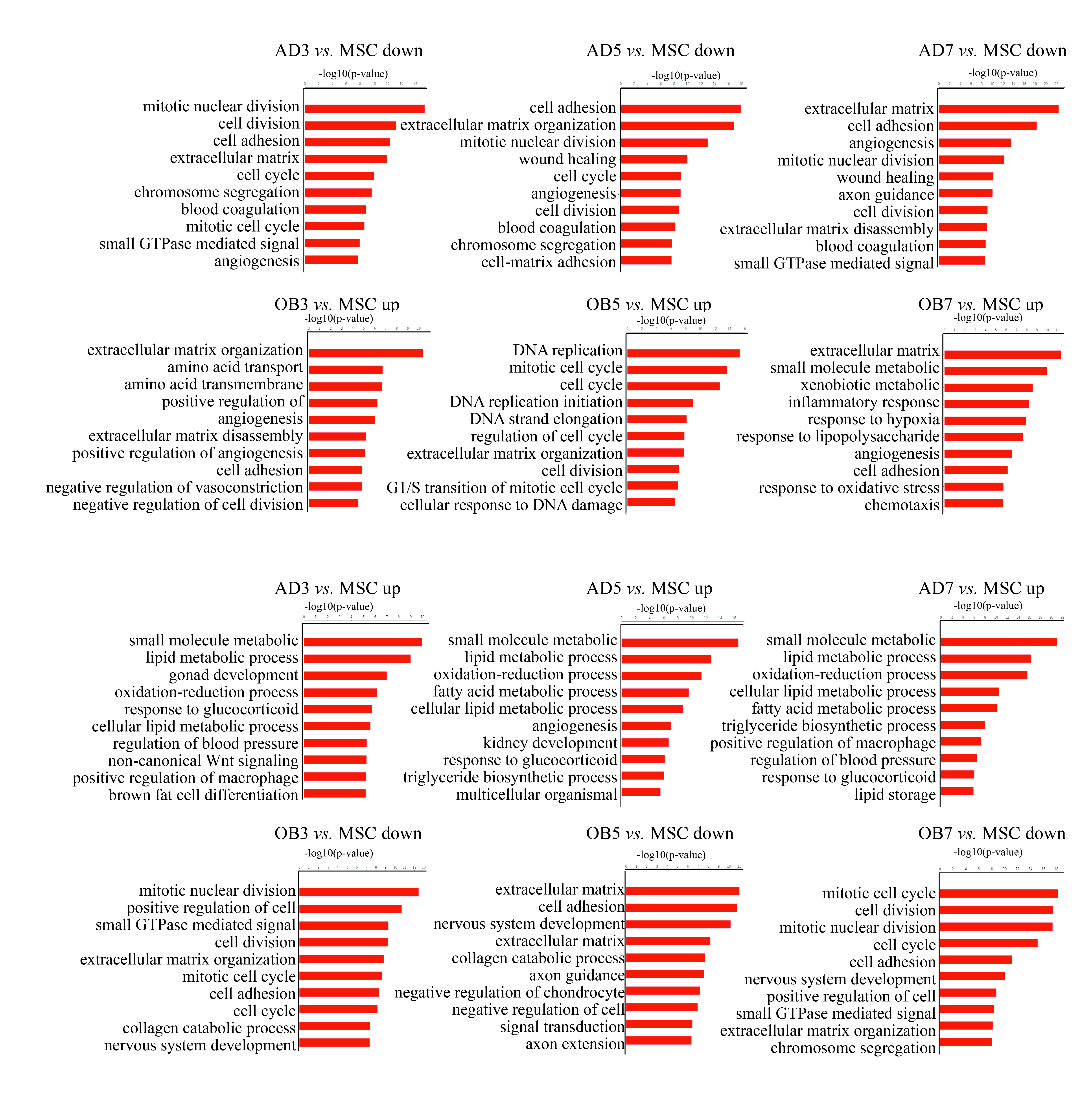

Supplement: Supplementary file 7 — Additional file 7: GO analysis of differentially expressed genes identified by RNA-seq. [file 12920_2022_1168_MOESM7_ESM.tif]

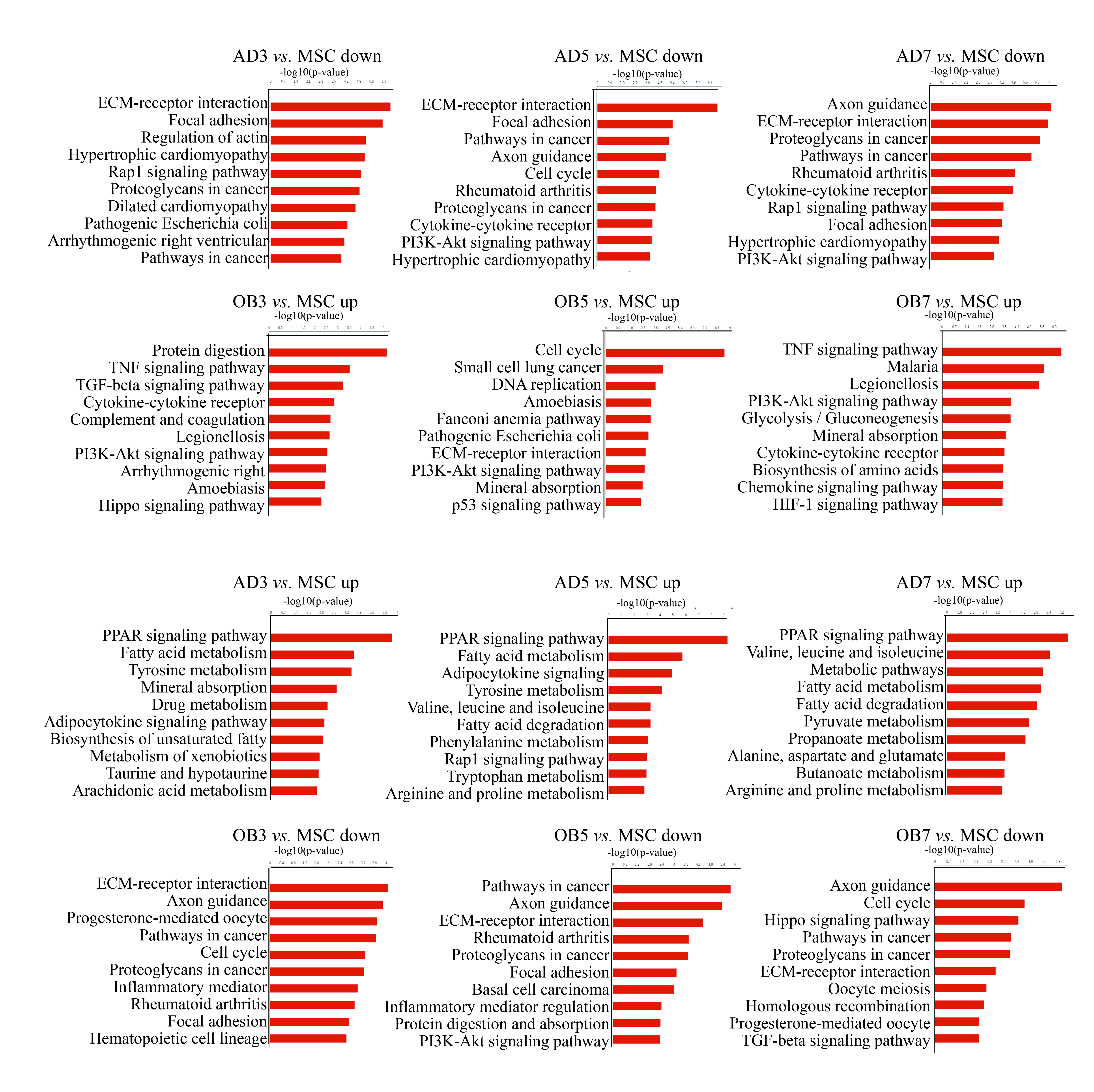

Supplement: Supplementary file 8 — Additional file 8: KEGG pathway analysis of differentially expressed genes identified by RNA-seq. [file 12920_2022_1168_MOESM8_ESM.tif]

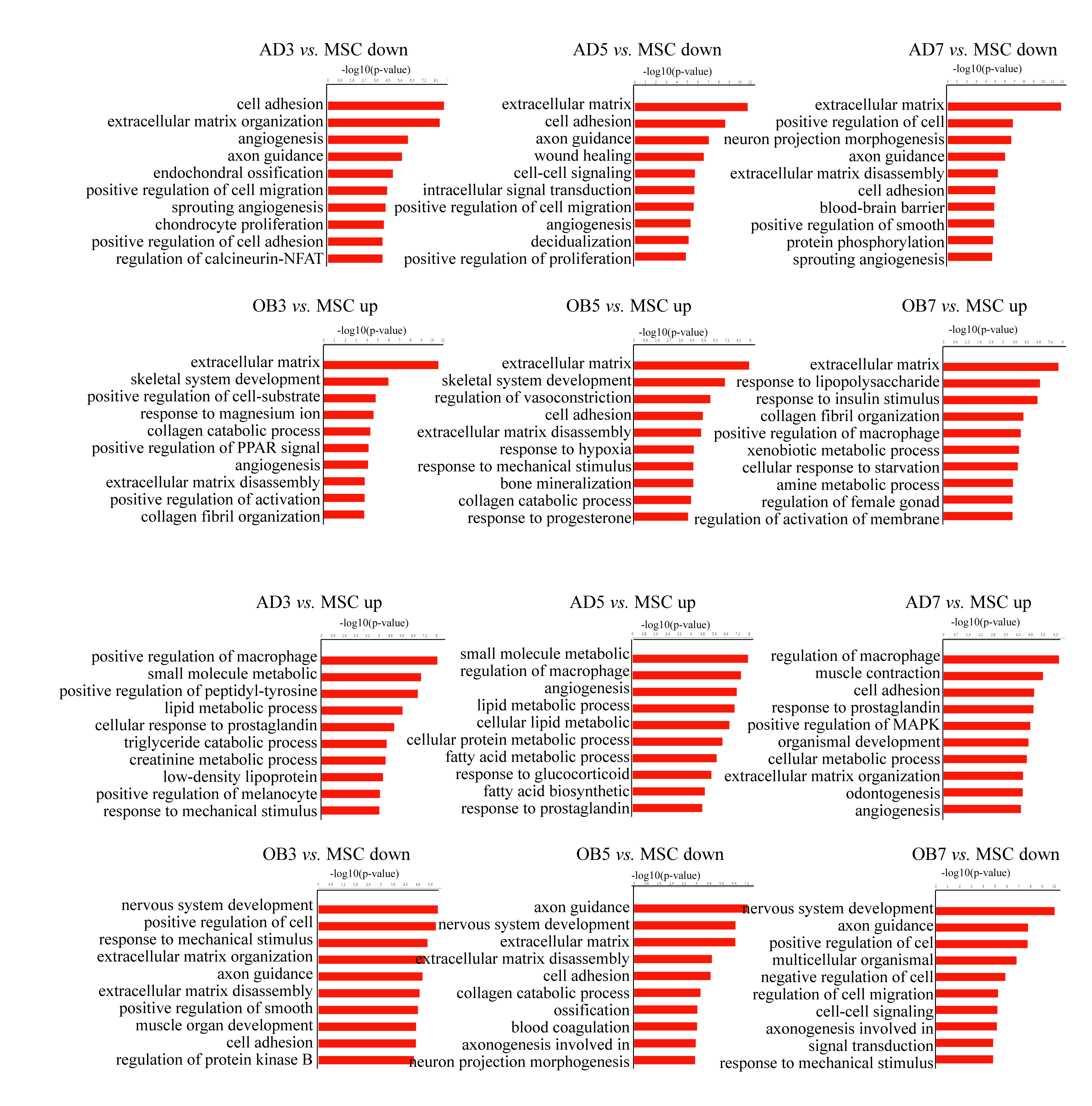

Supplement: Supplementary file 9 — Additional file 9: GO analysis of overlapped genes from ATAC-seq and RNA-seq data. [file 12920_2022_1168_MOESM9_ESM.tif]

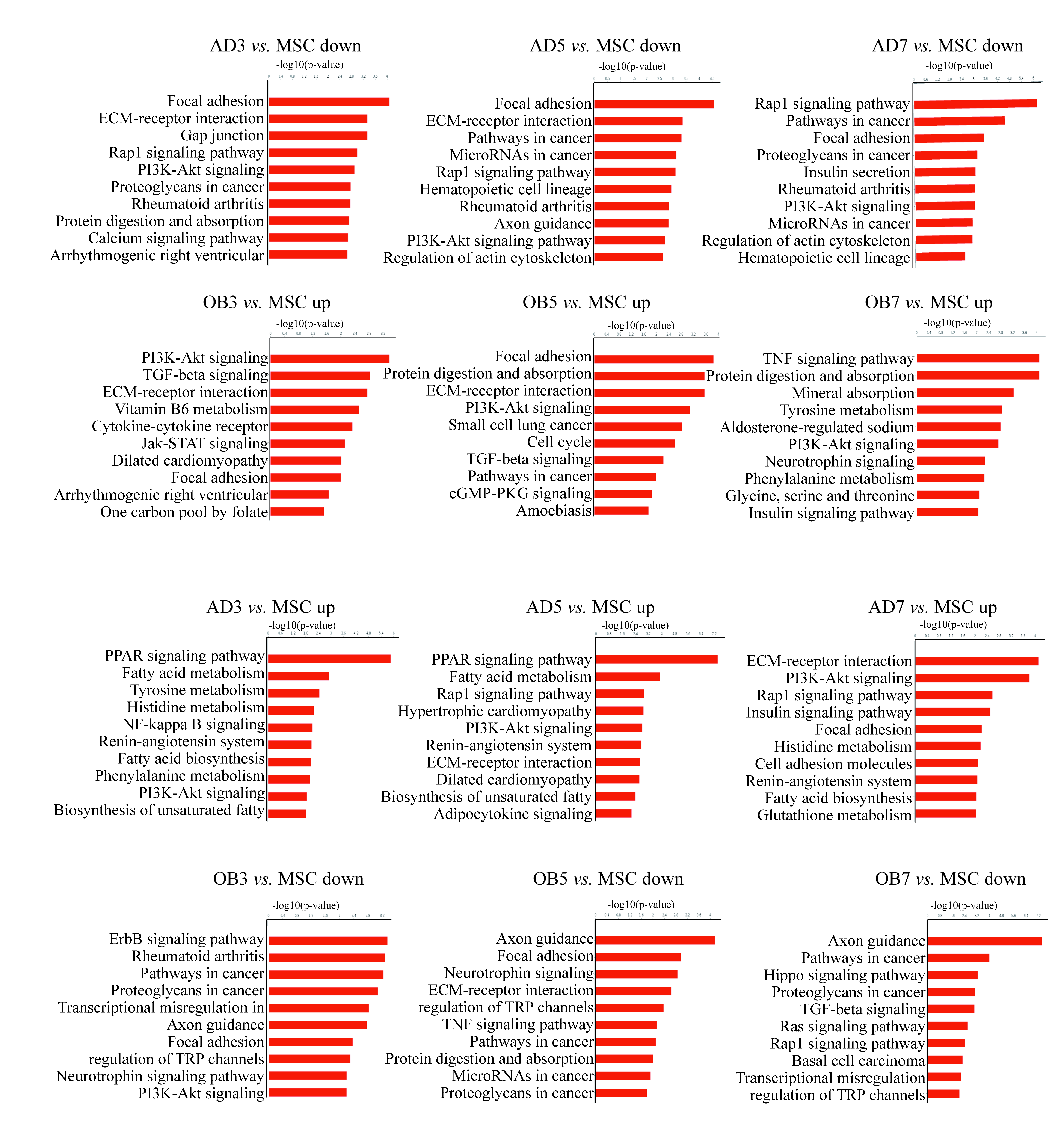

Supplement: Supplementary file 10 — Additional file 10: KEGG pathway analysis of overlapped genes from ATAC-seq and RNA-seq data. [file 12920_2022_1168_MOESM10_ESM.tif]
