# Supplementary material for: Alterations in chromatin accessibility during osteoblast and adipocyte differentiation in human mesenchymal stem cells
Source: BMC Med Genomics. 2022 Jan 31;15:17. doi: 10.1186/s12920-022-01168-1 (PMC8802426; doi:10.1186/s12920-022-01168-1)
Supplement: Supplementary file 3 — Additional file 3: The top 10 major motifs identified in each differentiated group. [file 12920_2022_1168_MOESM3_ESM.docx]

Supplementary Table 2. Top 10 motifs enriched in each group

| Rank | MSC | AD3 | AD5 | AD7 | OB3 | OB5 | OB7 |
| --- | --- | --- | --- | --- | --- | --- | --- |
| 1 | Fra1 | Fra1 | Fra1 | Fra1 | Fra1 | Fra1 | Fra2 |
| 2 | Atf3 | Fra2 | Fra2 | Fra2 | Atf3 | Fra2 | Fra1 |
| 3 | Fra2 | JunB | JunB | Atf3 | Fra2 | Atf3 | CTCF |
| 4 | JunB | Atf3 | Atf3 | JunB | BATF | JunB | Fosl2 |
| 5 | BATF | BATF | BATF | BATF | JunB | BATF | JunB |
| 6 | AP-1 | Fosl2 | Fosl2 | AP-1 | AP-1 | AP-1 | Atf3 |
| 7 | Fosl2 | AP-1 | AP-1 | Fosl2 | Fosl2 | Fosl2 | Jun-AP1 |
| 8 | Jun-AP1 | Jun-AP1 | Jun-AP1 | Jun-AP1 | Jun-AP1 | Jun-AP1 | AP-1 |
| 9 | Bach2 | CTCF | CTCF | Bach2 | CTCF | CTCF | BATF |
| 10 | CTCF | Bach2 | Bach2 | RUNX1 | Bach2 | Bach2 | BORIS |
