# Supplementary material for: Alterations in chromatin accessibility during osteoblast and adipocyte differentiation in human mesenchymal stem cells
Source: BMC Med Genomics. 2022 Jan 31;15:17. doi: 10.1186/s12920-022-01168-1 (PMC8802426; doi:10.1186/s12920-022-01168-1)
Supplement: Supplementary file 5 — Additional file 5: GO analysis of genes associated with accessible chromatin region. [file 12920_2022_1168_MOESM5_ESM.docx]

|  | MSC | AD3 | AD5 | AD7 | OB3 | OB5 | OB7 |
| --- | --- | --- | --- | --- | --- | --- | --- |
| 1 | phosphorylation | phosphorylation | phosphorylation | phosphorylation | phosphorylation | phosphorylation | phosphorylation |
| 2 | protein phosphorylation | regulation of transcription, DNA-templated | protein phosphorylation | regulation of transcription, DNA-templated | protein phosphorylation | protein phosphorylation | protein phosphorylation |
| 3 | positive regulation of transcription from RNA polymerase II promoter | protein phosphorylation | positive regulation of transcription from RNA polymerase II promoter | metabolic process | positive regulation of transcription from RNA polymerase II promoter | metabolic process | metabolic process |
| 4 | metabolic process | transcription, DNA-templated | metabolic process | transcription, DNA-templated | metabolic process | transport | regulation of transcription, DNA-templated |
| 5 | negative regulation of transcription from RNA polymerase II promoter | positive regulation of transcription from RNA polymerase II promoter | transport | apoptotic process | regulation of transcription, DNA-templated | protein transport | transcription, DNA-templated |
| 6 | positive regulation of apoptotic process | apoptotic process | axon guidance | gene expression | positive regulation of transcription, DNA-templated | intracellular signal transduction | protein transport |
| 7 | small molecule metabolic process | positive regulation of transcription, DNA-templated | regulation of transcription, DNA-templated | protein phosphorylation | endocytosis | endocytosis | positive regulation of transcription from RNA polymerase II promoter |
| 8 | intracellular signal transduction | metabolic process | positive regulation of Rho GTPase activity | negative regulation of transcription from RNA polymerase II promoter | protein transport | neurotrophin TRK receptor signaling pathway | cell cycle |
| 9 | axon guidance | organelle organization | angiogenesis | protein transport | intracellular signal transduction | small GTPase mediated signal transduction | negative regulation of transcription from RNA polymerase II promoter |
| 10 | transport | protein transport | brain development | positive regulation of Rho GTPase activity | transport | positive regulation of cell migration | positive regulation of transcription, DNA-templated |

Supplementary Table 3. GO analysis of genes associated with accessible chromatin region
